# F-Classify: Fuzzy Rule Based Classification Method for Privacy Preservation of Multiple Sensitive Attributes

**DOI:** 10.3390/s21144933

**Published:** 2021-07-20

**Authors:** Hasina Attaullah, Adeel Anjum, Tehsin Kanwal, Saif Ur Rehman Malik, Alia Asheralieva, Hassan Malik, Ahmed Zoha, Kamran Arshad, Muhammad Ali Imran

**Affiliations:** 1 Department of Computer Sciences, COMSATS University, Islamabad 45550, Pakistan; hasina.attaullah12@gmail.com (H.A.); adeel.anjum@comsats.edu.pk (A.A.); tehseenkanwal@yahoo.com (T.K.); 2 Department of Computer Science and Engineering, Southern University of Science and Technology, Shenzhen 518055, China; aasheralieva@gmail.com; 3 Cybernetica AS, 12618 Tallinn, Estonia; 4 Department of Computer Science, Edge Hill University, Lancashire L39 4QP, UK; malikh@edgehill.ac.uk; 5 James Watt School of Engineering, University of Glasgow, Glasgow G12 8QQ, UK; Ahmed.Zoha@glasgow.ac.uk (A.Z.); Muhammad.Imran@glasgow.ac.uk (M.A.I.); 6 College of Engineering and IT, Ajman University, Ajman 20550, United Arab Emirates; k.arshad@ajman.ac.ae; 7 Artificial Intelligence Research Centre (AIRC), Ajman University, Ajman 20550, United Arab Emirates

**Keywords:** DCP, F-Classify, membership function, MSA, MST, (p, k) angelization, QT

## Abstract

With the advent of smart health, smart cities, and smart grids, the amount of data has grown swiftly. When the collected data is published for valuable information mining, privacy turns out to be a key matter due to the presence of sensitive information. Such sensitive information comprises either a single sensitive attribute (an individual has only one sensitive attribute) or multiple sensitive attributes (an individual can have multiple sensitive attributes). Anonymization of data sets with multiple sensitive attributes presents some unique problems due to the correlation among these attributes. Artificial intelligence techniques can help the data publishers in anonymizing such data. To the best of our knowledge, no fuzzy logic-based privacy model has been proposed until now for privacy preservation of multiple sensitive attributes. In this paper, we propose a novel privacy preserving model *F-Classify* that uses fuzzy logic for the classification of quasi-identifier and multiple sensitive attributes. Classes are defined based on defined rules, and every tuple is assigned to its class according to attribute value. The working of the F-Classify Algorithm is also verified using HLPN. A wide range of experiments on healthcare data sets acknowledged that F-Classify surpasses its counterparts in terms of privacy and utility. Being based on artificial intelligence, it has a lower execution time than other approaches.

## 1. Introduction

In the digital era, data collection and storage for ultimate analysis are constantly expanding. The ownership of collected data allows data holders to utilize it for useful data mining. Given that data proprietors are not usually data professionals, collected data must be made accessible so that data analysts may use it. When data is shared for mutual benefit, individual privacy becomes a major concern. Individual privacy is compromised by the information set obtained, which comprises explicit identifiers, quasi-identifiers (QIs), sensitive attributes (SAs), and insensitive attributes. Personal identifiers, such as a name or a national identification number, are examples of explicit identifiers that are almost always re-identified. The privacy-preserving strategies presented in the literature [1,2,3] usually eliminated them from data sets. QIs are such attributes that, when combined, can assist to link a person to an externally available source, such as age, gender, and zip code. SAs contain sensitive information about a person, and their disclosure could significantly contribute to individual privacy. Insensitive attributes remain unchanged, as they are commonly not linked with privacy threats.

Today’s health care and other micro-data publishing entities are concerned about maintaining privacy without losing information. According to the research, there is an inverse relationship between privacy and information loss (data utility). K-anonymity [1] and its derivatives l-diversity [2], t-closeness [3] and many other [4,5] are examples of early work on privacy preservation based on generalization. The majority of the methods proposed in the literature [1,2,3,4,5,6] focus on single sensitive attribute data sets and rely on single-dimensional generalization. However, in most cases, real-world data publishing entities will have multiple sensitive attributes (MSAs). Known anonymization techniques like k-anonymity [1,6] and l-diversity [2] that were previously proposed do not preserve privacy for MSAs. In the case of MSAs, these techniques fail to protect privacy because the adversary breaches privacy with some background and non-membership knowledge attack.

### Motivation

In this section, different scenarios are presented to demonstrate how previous techniques fail to deal with MSAs. In Table 1, Gender, Age, and Zipcode are QIs whereas Disease, Treatment, Physician, Symptom, and Diagnostic method are SAs. Table 2 displays anonymization of the micro-data of Table 1 by removing the explicit identifiers from Table 1 and also showing generalization of QIs by making each group three diverse and four anonymous. The privacy breaches are explained in different scenarios.

Scenario 1: Let us start with a scenario in which the adversary already knows something about their next-door neighbor Richard. The adversary knows Richard is a 26-year-old man who lives in the same neighborhood as him, therefore he also knows their zip code. He noticed that Richard has recently lost weight. With such background and demographic knowledge, the adversary identifies from Table 2 that Richard belongs to group 1, and then discovers that the only patient in group 1 who has lost weight has cancer. In this manner, privacy is compromised by exploiting some demographic and background knowledge.

Scenario 2: In a different scenario, if the adversary from Table 2 knows Ana’s diagnostic method is an ELISA test, the adversary will be able to easily determine that Ana has HIV. As a result, existing approaches for single sensitive attributes are insufficient when it comes to preserving privacy for multiple sensitive attributes.

Scenario 3: In the case of MSAs, the previously proposed techniques for MSAs [7,8] still have some limitations. Single dimensional generalization is used in proposed MSA approaches, and there is a trade-off between privacy disclosure and data utility. SLOMS [9] has a demographic knowledge attack and significant information loss, whereas SLASMA [7] has a privacy risk from a demographic knowledge attack as well as low data utility.

Scenario 4: The approach (p, k) angelization [8] is similar to the strategy angelization [10], except that an adversary uses background knowledge to obtain a single SA value for each attribute by iteratively intersecting MSAs in correlated buckets. Additionally, because (p, k) angelization is based on MSA weight computations, the algorithm is more complex and takes longer to complete..

In this article, a fuzzy logic [11] based approach is proposed to address the limitations of previously proposed techniques; it is multi-dimensional partitioning and a rule-based technique. To preserve privacy, it offers multi-dimensional partitioning for both QIs and SAs. In the literature, fuzzy-based techniques for privacy preservation are proposed in [12,13], but none of them include MSAs.

**Figure 1:**
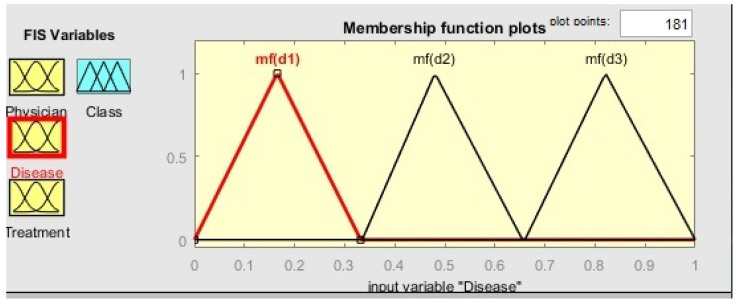
Matlab simulation of membership functions.

**Figure 2:**
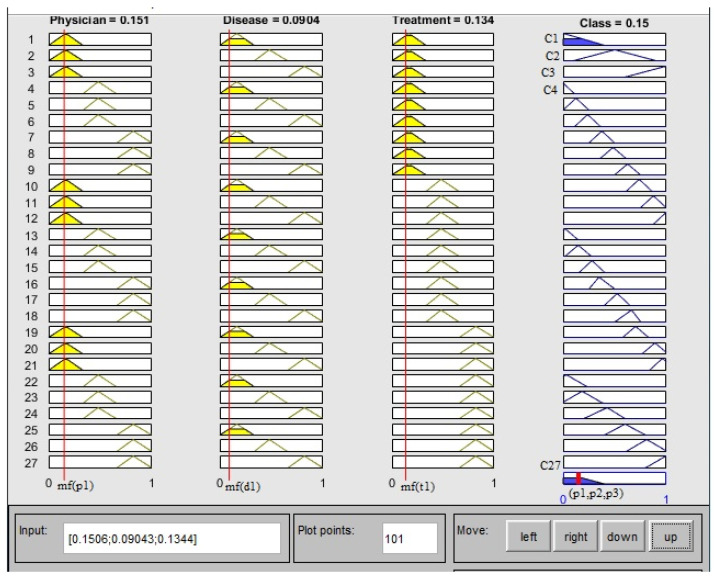
Matlab simulation of fuzzy logic, rules evaluation for Physician, Disease and Treatment.

The initial step in the proposed approach is to apply fuzzy classification on QIs (Age–Zipcode) and generate classes. Classification is not limited to 2-anonymous (two tuples in one class) or 3-anonymous (three tuples in one class); instead, each class has a different number of tuples. For example, class q-C2 has five patients in Table 3a, whereas class q-C4 has only one. SAs are classified after QIs have been classified. In SA classification, the class containing one patient is merged with another class to make the classification at least 2-anonymous and to avoid identity disclosure. In Figure 1 and Figure 2, Matlab [14] simulations of fuzzy logic membership functions (mfs) and rules assessment are shown. Table 3b shows the results of the fuzzy classification of three SAs.

As a final step, a permutation is used to generate anonymized data based on Table 3a–c. Table 4b,c are anonymized tables for MSTs to be published after patient identities were removed from Table 3b,c.

To evaluate the privacy breach of anonymized Table 4a, in the worst-case scenario, there is only one patient P12 in the last tuple of Table 4a, and the likelihood of re-identification is high. C1 and αC3 are the classes allocated to patient P12 based on SAs, and when looking at Table 4b,c, it can be seen that C1 has four tuples and αC3 has two. Going deeper into C1, there are three different diseases for disease attribute {Asthma, Flu, Indigestion}, as well as three distinct physicians {Suzan, Anas, Jem}. Similarly, there are two separate symptoms in αC3 {eating disorder, heartburn}, as well as three different diagnostic methods {Methacholine challenge Tests, Body mass index, Chest X-ray}. Going into further detail with C1, there are three different diseases {Asthma, Flu, Indigestion} for disease attribute and three different physicians {Suzan, Anas, Jem}. Likewise, in αC3 there are 2 different symptoms {eating disorder, heartburn} and 3 different diagnostic methods {Methacholine challenge Tests, Body mass index, Chest X-ray}. As a result, it would be difficult for an adversary to deduce a direct relationship between any information in a high-dimensional data set with only one attribute in one class.

The following are the main contributions of this paper:

- The article presents a fuzzy logic classifier (F-Classify) based on artificial intelligence (AI). The suggested methodology classifies QIs and SAa using a single methodology, namely fuzzy classification, rather than utilizing two distinct approaches for QIs and SAa. Instead of fixed classes/buckets, variable numbers of classes/buckets (k is variable) are formed in the proposed methodology.
- The proposed algorithm is verified for correctness using higher-level Petri nets (HLPN).
- The proposed F-classify approach is implemented in Python, and the results are compared to those obtained through (p-k) angelization. The results indicate that fuzzy classification (multi-dimensional partitioning) of correlated attributes increases data utility while permutation of multiple tables improves privacy. When compared to techniques that propose two different methods for QIs and SAs privacy, F-classify uses fuzzy logic for both QAs and SAs, resulting in minimal overhead.

In this article, the privacy preservation of MSAs is studied and proposed, the following section will highlight relevant work. Section 3 will discuss preliminaries and definitions, and Section 4 will describe the proposed work. Validation of the proposed approach is demonstrated using HLPN in Section 5. In Section 6, the findings and discussion are presented. The article concludes with Section 7.

## 2. Literature Review

This section summarizes the work that has been done so far in the area of privacy-preserving data publishing of single and multiple sensitive attribute data sets. Many privacy-preserving approaches have been proposed, using generalization, bucketization, or slicing techniques. K-anonymity [1,6], l-diversity [2], t-closeness [3] and many others [4,5,15,16,17] used generalization to provide privacy. Work based on bucketization has been proposed in [18,19,20,21], while slicing is a relatively new and evolving approach, first proposed by Li et al. [22].

The majority of these approaches focused on a single sensitive attribute. In reality, the data could be comprised of MSAs. The preservation of MSA privacy is still in its early phases, and a wide range of anonymization models have been presented in this regard, using several methodologies. Slicing [9,22], a method for anonymizing MSAs, was first presented in [22] for anonymizing high dimensional data. Because generalization causes information loss, Susan et al. [7] suggested a privacy model SLAMSA that utilized anatomization with slicing to resolve the information loss issue. Some enhanced slicing models, such as suppression and mondrian slicing, have been introduced in [23]. To eliminate the co-relationship between MSAs, Ref. [9] proposed “SLOMS”, which used slicing. Aside from slicing, numerous alternative approaches based on clustering and multi-sensitive bucketization have been proposed (MSB). For the privacy preservation of numerical MSAs, MSB-based approaches have been introduced [24], but these approaches have ignored textual data.

Another strategy (α, l) was proposed to meet the diversity of MSAs. Positive and negative disclosure risks are minimized in this technique by analyzing the correlation between MSAs [22,25,26]. (α, l) is also utilized in [27], together with anatomy, generalization, and suppression, which resulted in significant data loss. Ref. [28] introduces a rating approach for MSAs; it generalizes the sensitive attribute values, increasing information loss and hence decreasing utility. Another approach, Ref. [28], is based on rating, and the rating used could be compromised via association rules. The decomposition-based technique and its extension decomposition plus improved l-diversity for MSAs [29,30]. In [31], “ANGELMS” have been proposed to anonymize MSAs using vertical partitioning. Ref. [32] proposes a (p+)-sensitive and t-closeness model for MSAs that meets t-closeness requirements for the published table.

The privacy model (p, k) angelization [8] has some significant advantages over others, but it still has certain shortcomings because weights have been calculated and allocated to SAs based on interdependence and sensitivity of sensitive attributes. Weights for SAs cannot be calculated in every case, and weight calculation increases execution time. Khan et al. [33] in (p, k) angelization identify the fingerprint correlation attack and suggest an improved (c, k)-anonymization technique. The innovative KCi-slice [34] is a KC-slice model enhancement with better privacy and utility requirements. The author of [35] proposed multiple security levels for different SAs values. The proposed method claims more utility, but requires more time to execute.

Until now, we have only discussed MSAs with single record data sets. In the literature, there is also some work done in multiple records together with MSA data sets. Ref. [36] proposes the first privacy model for 1:M and MSAs, which evaluates the work of [8] for 1:M and MSAs-based privacy disclosures. Although the proposed approach provides good protection against adversarial attacks, it appears that efficiency can be improved. Recent work on adversarial attack identification in a balanced p sensitive k-anonymity based privacy model for 1:M and MSAs have been proposed. They presented 1:M MSA-(p, l)-diversity in [37] as an efficient, resilient, and utility aware privacy technique. Table 5 highlights some of the work proposed for MSAs.

To aggregate attributes based on QIs or MSAs, previously proposed models used techniques such as generalization, bucketization, and slicing. Information is lost while using the generalization approach provided in k-anonymity [1,2] since the records in one group are quite close to each other. Furthermore, because each attribute is generalized independently, there is no link between them. As a result, when analyzing the data, it is possible to find every potential combination of attributes. Despite the fact that bucketization has better utility than generalization [18,19,20,21], membership disclosure attacks are likely to occur because most bucketized algorithms use the same QIs values as the original table. Slicing of the data set has mostly focused on horizontal and vertical slicing. Slicing is mostly used for sensitive attributes, while QIs are either ignored or generalized via k-anonymity.

The basic terminologies and definitions used in this approach, as well as previously proposed approaches, will be highlighted in the following section.

## 3. Preliminaries

The techniques discussed earlier are based on single-dimensional generalization, and each technique provides a separate method for preserving QI and SA privacy. Single dimensional generalization is used to preserve QIs, while bucketization, slicing, and other techniques are used to preserve SAs. To maintain privacy, the approach used in this paper is based on fuzzy logic to provide multi-dimensional partitioning for both QIs and SAs. The basic terminologies and definitions used in this article and related articles are discussed in this section to help understand the presented methodology.

### 3.1. Notation

The data set is in the form of a table T with *m* data attributes and *n* tuples. The *m* data attributes are quasi-identifiers QI = $\left\{qi_{1},\ldots ,qi_{n}\right\}$ and sensitive attributes SA = $\left\{sa_{1},\ldots ,sa_{n}\right\}$. Adversary commonly uses sensitive attributes to reveal private information about individuals, and QI attributes can be linked to any other external data set to identify individuals. Table 6 lists the other notations used in this paper.

(Demographic knowledge attack) [8] If any individual *i* is uniquely recognized in any group *G* with *n* tuples through QIs, it means that the attacker uses demographic knowledge (dk). The adversary’s ability to find an individual [41] is facilitated by the individual’s QI attributes. If the attacker can trace an individual’s personal information via QIs, he is capable of launching a dk attack.

### 3.2. (p, k) Angelization Revisited

((p, k)-Angelization) [8] For a Table T, a batch partitioning = $\left\{B_{1},B_{2}\ldots B_{g}\right\}$ and a bucket partitioning = $\left\{C_{1},C_{2},\ldots C_{f}\right\}$ is given, a (p, k)-Angelization of Table T yields two different tables, sensitive batch table (SBT), and generalized table (GT).

### 3.3. Fuzzification

The process of fuzzification is the transformation of a precise number into a fuzzier one. In this step, inputs are changed into linguistic variables that can be used with fuzzy sets. Below are definitions of linguistic variables, membership functions (mfs), and fuzzy sets.

Linguistic variable: Let Table T be the universe under consideration with m data attributes and n tuples. Table T has crisp data. The first step in mapping crisp data into fuzzy data is to define linguistic variables (lv). The lv is a data attribute with some values that can include QIs and SAs. In Table 1, QI age is a linguistic variable and it has some linguistic value, i.e., 27 years.

Membership function (mfs): The degree of membership is determined by the values of linguistic variables. The value assigned to attributes is called its degree. Mfs are determined based on degree of membership. Depending on the values of the linguistic variable, Mfs can be two, three, or four. For example, we can define two mfs for the linguistic variable age in Table 1 as $\mu_{1}$ = (25–33 years) and $\mu_{2}$ = (34–48 years).

Fuzzy sets: Mfs are used to generate fuzzy sets. The fuzzy sets include everything between completely false (0.0) and completely true (1.0). For example, the two mfs for age *A* = $\mu_{1}$, $\mu_{2}$ form one fuzzy set. Assume that Table T is the universe under consideration and *t* is a specific element of *T*, then *A* is a fuzzy set defined on *T* and can be expressed as;

(1) $$\begin{array}{c} A=\{\left(t,\mu_{A}\left(t\right)\right),t\in T\}\\ \mu_{A}\left(t\right)=T\to \left[0,1\right] \end{array}$$

$\mu_{A}$ is called the membership function.

Logical operations on fuzzy sets: The fuzzy set theory comprises the operations union, intersection, complement, and inclusion, just like classical set theory. In fuzzy logic, the various logical operations for compound statements in Equation (1) are considered as implications [12,32]. The implication used in the proposed approach is UNION or AND, which is defined as follows in terms of characteristic functions. (UNION) Union of two fuzzy sets *A* and *B* using (1) will be:

(2) $$\mu_{\left(A\cup B\right)}\left(t\right)=AND\left[\mu_{A}\left(t\right),\mu_{B}\left(t\right)\right]$$

Fuzzy Inference: The fuzzy relation R=A→B is used to represent a fuzzy rule. *R* can be considered as a two-dimensional membership function for a fuzzy set (1). By applying implication as a union using Equation (2) and employing IF-THEN rules, a fuzzy inference engine is formed. Possible rules are calculated based on linguistic variables and membership functions. Total rules can be calculated as ${\left(\mu \right)}^{lvs}$.

Defuzzification: Following the evaluation of the rules, the input fuzzy sets are defuzzified, resulting in a set of crisp output values. The center of gravity (CoG) defuzzification method is used to defuzzify the fuzzy system [42]. In CoG, values are taken from the inference engine and aggregated.

### 3.4. HLPN

HLPN is used to verify the correctness of an algorithm. The HLPN [43] is a 7-tuple with N = (P, T, F, φ, Rn, L, $M_{0}$). F, P, and T belong to a dynamic structure, whereas L, φ, and Rn reflect static semantics in the group of 7-tuples. The P represents a finite set of places, each of which represents a single part of the system. T denotes the set of finite transitions, where transitions represent the system’s variations. Rn explains the transition rules, L signifies a label on F, and $M_{0}$ denotes the initial marking in the 7-tuple definition of HLPN.

## 4. Proposed Approach: F-Classify

Section 2 investigates previously proposed techniques for single sensitive attributes and multiple sensitive attributes. According to the findings, privacy breaches occur when single sensitive attribute-based approaches are applied to MSAs. Section 2 discusses and compares MSA-based techniques based on privacy violations and utility requirements. There appears to be a trade-off between privacy and utility in the majority of MSA-based approaches. To reduce such trade-offs, this article introduces F-Classify, an AI-based classification methodology for QIs and MSAs. F-Classify will publish several tables. One anonymized QIs table and numerous MSAs tables will be published. The number of MSAs tables is determined by the number of sensitive attributes in micro-data. In the following sections, we will go through how F-Classify works.

### 4.1. Linguistic Variables and Fuzzy Sets

The first step is to convert crisp input (m data attributes) to linguistic variables. The values for lvs (QIs and SAs) are specified first, and then the mfs are generated. For both numerical and categorical attributes, the criteria for defining mfs are different.

- For numerical attributes sort the data in any order, then divide it into two/three/four (depending on mfs) equal lists.
- For categorical attributes, select unique attribute values from the list. Then, for each distinct attribute, assign a random number between 0 and 1. After assigning a random number, divide the unique list into two/three/four equal lists using the same technique.

Let *lv(q)* denote the linguistic variable for QIs, and *lv(sa)* denote the linguistic variable for sensitive attributes. First, define mfs, then fuzzy sets for lv(q) and lv(sa). Equations (3) and (4) show fuzzy sets for linguistic variable QIs and MSAs, respectively.

(3) $$\begin{array}{c} QI_{A}\left(lv\left(q_{A}\right)\right)=\left\{lv\left(q_{A1}\right),\;lv\left(q_{A2}\right),\;\ldots ,\;lv\left(q_{Ai}\right)\right\}\\ QI_{B}\left(lv\left(q_{B}\right)\right)=\left\{lv\left(q_{B1}\right),\;lv\left(q_{B2}\right),\;\ldots ,\;lv\left(q_{Bi}\right)\right\} \end{array}$$

(4) $$\begin{array}{c} SA_{a}\left(lv\left(sa_{a}\right)\right)=\left\{lv\left(sa_{a1}\right),\;lv\left(sa_{a2}\right),\;\ldots ,\;lv\left(sa_{ai}\right)\right)\}\\ SA_{b}\left(lv\left(sa_{b}\right)\right)=\left\{lv\left(sa_{b1}\right),\;lv\left(sa_{b2}\right),\;\ldots ,\;lv\left(sa_{bi}\right)\right)\}\\ SA_{c}\left(lv\left(sa_{c}\right)\right)=\left\{lv\left(sa_{c1}\right),\;lv\left(sa_{c2}\right),\;\ldots ,\;lv\left(sa_{ci}\right)\right)\} \end{array}$$

Output variables: Based on QAs and SAs, the output will be a crisp value. Output classification is in (5) for two attribute QAs and three MSAs.

(5) $$\begin{array}{c} QI_{A}-QI_{B}\left(q-C\right)=\left\{q-C_{1},\;q-C_{2},\;\ldots ,\;q-C_{\alpha }\right\}\\ SA_{a}-SA_{b}-SA_{c}\left(sa-C\right)=\left\{sa-C_{1},\;sa-C_{2},\;\ldots ,\;sa-C_{\alpha }\right\} \end{array}$$

### 4.2. Fuzzy Inference Rule-Based

Section 4.1 defined linguistic variables and membership functions; the next step is to define rules based on fuzzy sets and implications. The number of rules will be determined by the number of lvs and the number of mfs. For any, *i* and *j* and QIs *A* and *B* rules will be calculated in (6).

(6) $$\begin{array}{c} if\;QI_{A}\;is\;lv\left(q_{Ai}\right)\;and\;QI_{B}\;is\;lv\left(q_{Bj}\right)\\ action=q-C_{\left(i+j-1\right)} \end{array}$$

For any, *i*, *j*, and *k* and SAs *a*, *b*, and *c* rules will be calculated in (7).

(7) $$\begin{array}{c} if\;SA_{a}\;is\;lv\left(sa_{ai}\right)\;and\;SA_{b}\;is\;lv\left(sa_{bj}\right)\;and\;SA_{c}\;is\;lv\left(sa_{ck}\right)\\ action=sa-C_{\left(i+j+k-2\right)} \end{array}$$

### 4.3. Defuzzification

Defuzzification is performed on the output of evaluated rules. As a result of defuzzification, we only get one tuple at a time. The result of defuzzification of SAs in the provided example is shown in Figure 2. In Figure 2, the selected attribute physician is p1, the disease is d1, and the treatment is t1, therefore the defuzzification result is class C1.

### 4.4. Permutation

Rules are used to classify QIs and MSAs. We assign these classes to tuples in the data set, and we now have multiple tables based on the classes we defined. Values from the SAs table are permuted into the QIs table to generate the anonymized table. The formal modeling and analysis of the F-Classify algorithm will be explained in the next section.

## 5. Formal Modeling and Analysis

The design and working of the F-Classify algorithm are described in depth in Section 4. Here, F-Classify algorithm formal modeling and analysis is performed using HLPN [43].

### 5.1. F-Classify Algorithm

The F-Classify algorithm is explained in depth in the following sections. Two parts make up the algorithm. Fuzzification is the first step, while permutation is the second.

The F-classify Algorithm 1 starts with splitting the table into QIs and SAs attributes subsets. Furthermore, it generates tables (QT) and (STm) with new attribute classes and attributes of sub-set tables for every data sub-set. Data attributes are initialized and called linguistic variables (lv). Line 1–3 in Algorithm 1 defines mfs for every lv, mfs can be 2, 3, or 4 for every attribute. Line 4: loop from 1 to the number of subsets (k). Line 5: loop from 1 to no of rules (η). Line 6: making rules for data-set from 1 to total rules. Line 7: Assign output class to each rule. Line 10: loop from 1 to the number of tuples (n). Line 11: loop from 1 to the number of rules (η). Line 12: condition to check, tuple from Table T belongs to which output class. Line 13: generate tables (QT) and (STm) with new attribute class and attributes of sub-set table for every data sub-set.
**Algorithm 1** F-Classify algorithm: Fuzzification.**Require:** Table T with *m* data attributes and *n* tuples**Ensure:** Release Table QT and $\left\{ST_{1},\ldots ,ST_{m}\right\}$*Initialisation* Split the table T into multiple sub-sets (One sub-set for QI and remaining sub-sets for SAs) from the data sets. *multiple sub-sets = d1, d2, d3…dk* *data attributes (linguistic variables) = $lv_{1},lv_{2},\ldots ,lv_{m}$ M*embership functions = μ *γ = number of attributes in one subset* *η = number of member-ship functions for lv* *α: Number of fuzzy rules ($\alpha =\eta^{\gamma }$)* *Rules R [] = $\left\{R_{1},\ldots ,R_{\alpha }\right\}$* *Classes [] = $\left\{C_{1},\ldots ,C_{\alpha }\right\}$* 1: **for** i=1 to *m* **do** 2:     define mfs for lvs 3: **end for** 4: **for** i=1 to *k* **do** 5:     **for** j=1 to η **do** 6:         R[i]←AND(lv[1][j],lv[2][j],…lv[γ][j]) 7:         C[i]←R[i] 8:     **end for** 9: **end for**10:**for**i=1 to *n* **do**11:    **for** j=1 to α **do**12:       **if** (T(Tuple)∈(C[α])) **then**13:          Generate a new table according to Classes for QIs (QT) and SA$\left(ST_{1},ST_{2},\ldots ST_{m}\right)$14:       **end if**15:    **end for**16:**end for**17:**return** Tables QT and $ST_{1},ST_{2},\ldots ,ST_{m}$

After tables are generated using the fuzzification procedure, the tables are passed to a permutation procedure to permute and get a higher level of privacy. Algorithm 2 takes tables from the fuzzification module and generates anonymize tables. Line 1-2: loop from 1 to the number of tuples (n), loop from 1 to the number of sub-sets (k). Line 3: condition to check QT(tuple) belongs to which class of every SAs subset table (ST). Line 4: append attribute class in QT for every subset of ST. Line 9: publish QT and SAs subsets tables $ST_{1},ST_{2},\ldots ,ST_{m}$.

In the following part, we will go through formal modeling and analysis.
**Algorithm 2** F-Classify algorithm: Permutation.**Require:** QT and $ST_{1},ST_{2},\ldots ST_{m}$ from procedure Fuzzification**Ensure:** Anonymize Table QT and Anonymize $MST_{1},MST_{2}\ldots MST_{m}$  **for** i=1 to *n* **do**2:      **for** j=1 to *m* **do**          **if** ($QT\left(Tuple\right)\in (ST_{m}$) **then**4:               Append attribute Class ($ST_{m}$) in QT for every ST         **end if**6:      **end for** **end for**8: **for** i=1 to *T* **do**     Release Anonymize Table QT and $MST_{1},MST_{2}\ldots MST_{m}$10:**end for****return** Anonymize Table QT and Anonymize $MST_{1},MST_{2}\ldots MST_{m}$

### 5.2. Formal Modeling and Analysis

**Figure 3:**
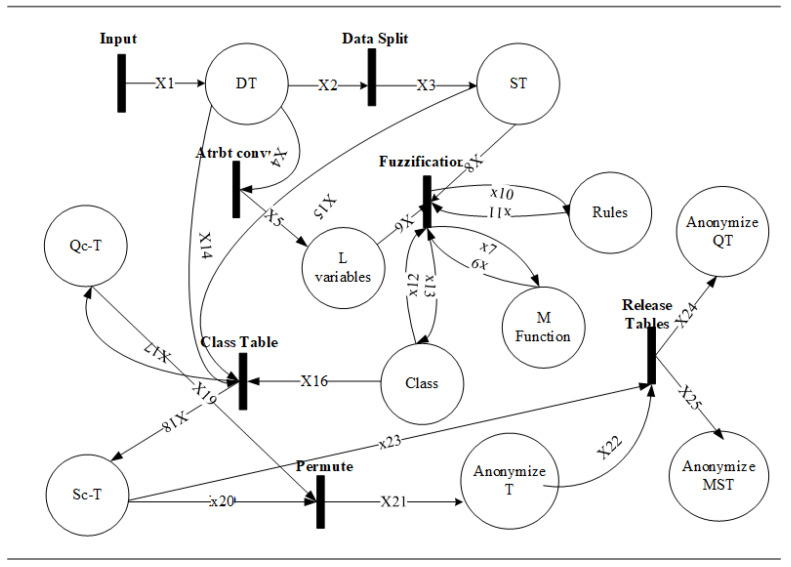
HLPN for F-Classify algorithm.

We formally validate the working of the F-Classify algorithm along with its properties. To achieve the formal analysis, HLPN and Z3 languages are used. The HLPN model has been transformed into SMT-Lib [44] together with the correctness properties to illustrate the correctness of the F-Classify algorithm. Properties are then executed via the Z3 solver to verify their correctness. In HLPN, the algorithm was first presented in terms of its mathematical properties. These attributes are first translated into SMT-Lib to see if they are valid, and then they are executed through the Z3 solver. In [44], the formal definitions of SMT and Z3 solver are presented. The notations used in this section are represented in Table 6. Figure 3 depicts the HLPN for the F-Classify algorithm. Table 7 defines the variable types that were used and their explanations. The places and descriptions included in the HLPN F-Classify algorithm are shown in Table 8. The transitions have been labeled as Input in Figure 3. The first Input transition is a raw data table with m attributes and n records of patient electronic health records (EHRs) stored in the place DT. In the input transition, a raw data table with m attributes and n records is given, which is subsequently separated into different QI and SAs subsets using the Dsplit function. In Equation (8), the entire data split procedure is shown. All *m* attributes are then translated into linguistic variables lv${}_{m}$ in (9).

(8) $$\begin{array}{c} R(\mathrm{Data}\mathrm{Split})=\forall i2\in x2,i3\in x3|\\ \left(i3\left[1\right],i3{\left[2\right]}_{i_{\forall i3[2]\in i}}\right):=DSplit\left(i2{\left[2\right]}_{m_{\forall i2[2]\in m}}\right)\wedge \\ x3^{{}^{\prime }}:=x3\cup \left\{i3\left[1\right],i3\left[2\right]\right\} \end{array}$$

(9) $$\begin{array}{c} R(\mathrm{Attrb}\mathrm{Conversion})=\forall i4\in x4,i5\in x5|\\ \left(i5{\left[1\right]}_{m_{\forall i5[1]\in m}}\right):=Conversion\left(i4{\left[1\right]}_{m_{\forall i4[1]\in m}}\right)\wedge \\ x5^{{}^{\prime }}:=x5\cup \left\{\left(i5\right)\right\} \end{array}$$

All membership functions η are defined for linguistic variables m. Now rules are formed based on the combined values of linguistic variables and membership functions as $\eta^{m}$ and saved in Rules, then output classes are assigned to each specific rule in (10) and (12).

(10) $$\begin{array}{c} R(\mathrm{Fuzzification})=\forall i6\in x6,i7\in x7,i10\in x10,\\ i11\in x11,i13\in x13|\\ \left(i7{\left[1\right]}_{m_{\forall i7[1]\in m}}\right):=Mfunction{\left(i6\left[1\right]\right)}_{m_{\forall i6[1]\in m}}\wedge \\ x7^{{}^{\prime }}:=x7\cup \left\{\left(i7\right)\right\}\wedge \\ \left(i10{\left[1\right]}_{n_{\forall i10[1]\in n}}\right):=Rule((i6\left[1\right]\left[\eta \right]\wedge \\ {i6\left[\gamma \right]\left[\eta \right])}_{n_{\forall i6[\gamma ][\eta ]\in n}})\wedge \\ x10^{{}^{\prime }}:=x10\cup \left\{\left(i10\right)\right\}\\ {\left(i13\left[1\right]\right)}_{n_{\forall i13[1]\in n}}:={\left(i11\left[1\right]\right)}_{n_{\forall i11[1]\in n}}\wedge \\ x13^{{}^{\prime }}:=x13\cup \left\{\left(i13\right)\right\} \end{array}$$

After that, each record in the data table is compared to check its class as depicted in (11), then we construct class-based quasi identifier table QcT (Table 3a) and multiple sensitive attribute tables ScT (Table 3b,c).

Now, from the class-based quasi identifier table QcT, it is checked whether the corresponding class is present in the sensitive attribute table ScT. Quasi identifier table QcT is appended to each class of multiple sensitive attribute tables ScT and saved in Anonymize T. After that, each record in the data table is checked as it is present in which class in (11), then we construct a class-based quasi identifier table QcT (Table 3a) and multiple sensitive attribute tables ScT (Table 3b,c).

It is then checked whether the matching class is present in which sensitive attribute table ScT, using the class-based quasi identifier table QcT. Each class of multiple sensitive attribute tables ScT has a quasi-identifier table QcT appended to it and saved in Anonymize T. Table 4a shows the anonymized form of the table. We publish anonymized QT (Table 4a) and MST (Table 4b,c) tables with multiple sensitive attributes. The above procedure is represented by the last transition Release Table, depicted in (13).

(11) $$\begin{array}{c} R(\mathrm{Class}\mathrm{Table})=\forall i14\in x14,i15\in x15,i16\in x16,\\ i17\in x17,i18\in x18|\\ ((i14[1]\in (i16[1])=TRUE))\to \\ \left(i17[1],i17[2],i17[3]):=Makeclasstable(i14[1\right]\\ \|\;i15[1]\;\|\;i16[1])\wedge \\ x17^{{}^{\prime }}:=x17\cup \left\{\left(i17\left[1\right],i17\left[2\right],i17\left[3\right]\right)\right\}\vee \\ ((i14[1]\in (i16[2])=TRUE))\to \\ \left(i18[1],i18[2],i8[3]):=Makeclasstable(i14[2]\;\|\;i15[2\right]\\ {\left\|\;i16\left[2\right]\right)}_{p_{\forall i16[2]\in p}}\wedge \\ x18^{{}^{\prime }}:=x18\cup \left\{\left(i18\left[1\right],i18\left[2\right],i18\left[3\right]\right)\right\} \end{array}$$

The working and properties of the F-Classify privacy-preserving model have been formally verified in this section. Multiple sensitive attributes are protected from membership, attribute, and identity disclosure while using a fuzzy logic-based classification methodology. In the following section, we will look at the performance of the proposed privacy model.

(12) $$\begin{array}{c} R(\mathrm{Permute})=\forall i19\in x19,i20\in x20|\\ (\left(i19\left[2\right]\in {\left(i20\left[2\right]\right)}_{i_{\forall i20[2]\in i}}\right)=TRUE)\to \\ \left(i21[1],i21[2],i21[3],i21[4]\right)\\ :=(i19[1],i19[2],i19[3])\;\|\;(i20[3])\wedge \\ x21^{{}^{\prime }}:=x21\cup \left\{i21\left[1\right],i21\left[2\right],i21\left[3\right],i21\left[4\right]\right\} \end{array}$$

(13) $$\begin{array}{c} R(\mathrm{Release}\mathrm{Table})=\forall i22\in x22,i23\in x23,\\ i24\in x24,i25\in x25|\\ (i24[1],i24[2],i24[3]):=(i22[1],i22[2],i22[3])\wedge \\ i25[1]:=i23[2]\wedge i25[2]:=i22[4]\\ \wedge x24^{{}^{\prime }}:=x24\cup \left\{i24\left[1\right],i24\left[2\right],i24\left[3\right]\right\}\\ \wedge x25^{{}^{\prime }}:=x25\cup \left\{i25\left[1\right],i25\left[2\right]\right\} \end{array}$$

## 6. Results and Discussion

This section compares and discusses the experimental results of the proposed methodology to those of previously suggested techniques.

### 6.1. Experimental Setup

Our model is implemented on an Intel Core i7-3520M computer with a 500 GB hard drive and 8 GB RAM, running Windows 7. Python is the programming language used in the implementation. The two data sets for experiments have been taken from the UCI repository http://archive.ics.uci.edu/ml/datasets/Heart+Disease (accessed on 10 June 2020) and https://archive.ics.uci.edu/ml/datasets/Adults (accessed on 10 August 2020). The data used are from the Hungarian Institute of Cardiology and Cleveland Clinic Foundation of Heart Disease and Adults. In the heart disease data set, there are almost 76 attributes, but we have used 14 attributes for experimental purposes. Furthermore, out of 14, 12 are SAs and 2 of them are QIs. The QIs in the data set are age and gender, and we have added another QI zipcode for experimental purposes. The 12 SAs used are cp (chest pain type), trestbps (resting blood pressure), chol (serum cholerstrol), fbs (fasting blood sugar), restecg (resting electrocardiographic results), thalach (maximum heart rate achieved), exang (exercise-induced angina), oldpeak (ST depression induced by exercise relative to rest), slope (the slope of the peak exercise ST segment), ca (number of major vessels), thal (type of defect) and num (diagnosis of heart disease). The value of cp is either 1, 2, 3, or 4 based on different pain conditions. The value of num is either 0 or 1, and the value of the slope is either 1 (upsloping), 2 (flat), or 3 (downsloping). We have used 284 records after removing missing values records. In the second adult data set a total of 40,000 tuples are taken for experimental purposes. The 12 attributes are selected for analysis, and out of 12, 9 are sensitive attributes and 3 are QIs (age, gender, and zip code). The comparative analysis based on different parameters has been done between the proposed model and (p, k) angelization.

### 6.2. Measurement of Privacy

We ca not quantify the privacy level or information leakage of any method scientifically, but we can calculate the probability of information leakage. In F-Classify, we have used fuzzy logic for classification or grouping of tuples so information leakage depends on membership functions defined for QIs and SA and class/group size. In F-Classify we have dynamic class/group size, so to measure privacy leakage we will sum the number of records in each group and then find the probability, P = $\frac{1}{\alpha }\times 100$, where α is the sum of records based on QIs and sensitive attributes in each class/group.

(14) $$\begin{array}{c} \alpha =\sum_{i}\left(k_{i}+s_{i}\right) \end{array}$$

**Figure 4:**
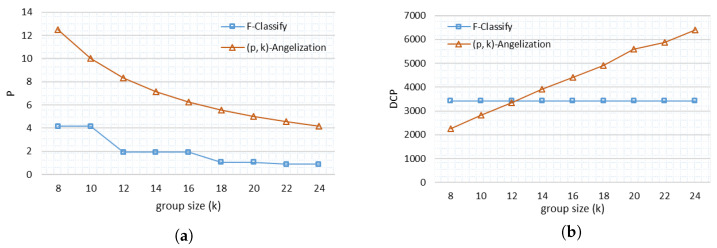
Comparison of Heart Disease Data set (**a**) Probability of re-identifying a record (**b**) Discernibility Penalty.

In (14), *k* is the number of records in each class/group based on QI and is denoted by $\left(k_{1},\ldots ,k_{i}\right)$, is the number of sensitive attributes in each class/group and *i* is the number of membership functions defined for QIs. We have multiple groups against one mf, therefore summation is used to add records from every group. For approach (p, k) angelization SA are not categorized, therefore the probability is based only on group size, as $P=\frac{1}{k}\times 100$, where *k* is a group size that is static and predefined. So this probability only depends on the number of records in one group. The likelihood of finding a record in the case of (p, k) angelization is substantially higher than in the case of F-Classify, as shown in Figure 4a. We have classification based on QI and SAs in F-Classify, therefore the chance of identifying a record in a data set is determined by the sum of records in each mf and the number of SA against each record. In (p, k) angelization, however, only the records in one group are considered. Furthermore, when the number of records in a data set increases, the group size gets bigger in F-Classify, and the likelihood of finding a record decreases, but this is not the case with (p, k) angelization, which has a fixed group size. The number of records in (p, k) angelization does not affect group size.

### 6.3. Discernibility Penalty

The Discernibility penalty (DCP) is a measure of indistinguishable records. Each record gets a penalty for being indistinguishable from other records, which is used to calculate DCP. The lower the DCP value, the more indistinguishable the records are from one another. If we have C indistinguishable classes, we can use (15) to determine the DCP.

(15) $$\begin{array}{c} DCP=\sum_{C}{\left|C\right|}^{2} \end{array}$$

We used fuzzy logic with multi-dimensional partitioning in our methodology, which fuzzifies and distinguishes records. We can observe in Figure 4b that DCP grows with increasing group size in the case of (p, k) angelization, but DCP remains the same in the case of F-Classify. We have different group sizes in one table in F-Classify, as seen in Table 3a. We have one tuple in one class/group and five tuples in the other class/group, giving us 1, 5, 6, and 1 group sizes in one table while maintaining DCP. As a result, DCP has a smaller value in F-Classify than (p, k) angelization, where DCP increases with the increase of group size.

### 6.4. Normalized Certainty Penalty (NCP)

**Figure 5:**
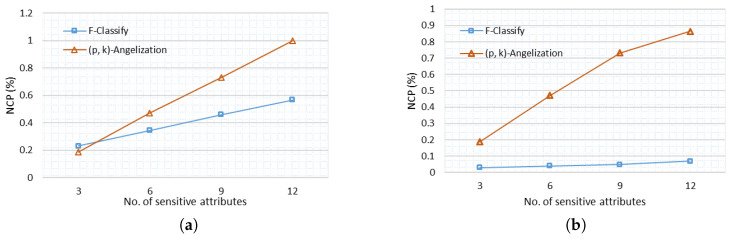
Normalized Certainty Penalty (NCP) (**a**) NCP Heart Disease (**b**) NCP Adults.

To check the utility of proposed techniques, the idea of certainty penalty (CP) is proposed by Xu et al. [5]. CP is a utility-based metric, it is helpful to get information loss, and it also illustrates the significance of every attribute. Normalized certainty penalty (NCP) is calculated using (16).

(16) $$NCP=\sum_{i}\sum_{j}\left(\frac{z_{ij}-y_{ij}}{A_{j}}\right)$$

where *y* and *z* are the range of tuples defined after classification of table T, and A is the attribute for which NCP is calculated. In Figure 5a, NCP is calculated for F-Classify and (p, k) angelization. NCP is calculated based on generalization steps in the case of (p, k) angelization and in F-Classify it is based on classification of attributes. Information loss is proportional to generalization; the more the generalization, the greater the information loss. The information loss in (p, k) angelization is more than in F-Classify, as seen in Figure 5a. When there are few sensitive attributes, information loss is greater in F-Classify, but it gradually decreases in comparison to (p, k) angelization as the number of sensitive attributes increases. NCP is quite low in F-Classify in the adults data set, as seen in Figure 5b. In terms of information loss, the suggested algorithm’s low NCP shows that it performs well with larger data sets.

### 6.5. Query Error

Query Error is another way to assess the suggested F-Classify utility. Comparing anonymized datasets aggregated query results [45,46] is one way to determine the efficiency of privacy models. Query error is computed using Equation (17).

(17) Query−Error=Estimated−count−Actual−count/Actual−count

The actual query count is the result of the query executed on the original data-set T, whereas the estimated query count is the count obtained by anonymizing the data-set (T*). The query accuracy results are compared to the number of groups and query dimensionality.

**Figure 6:**
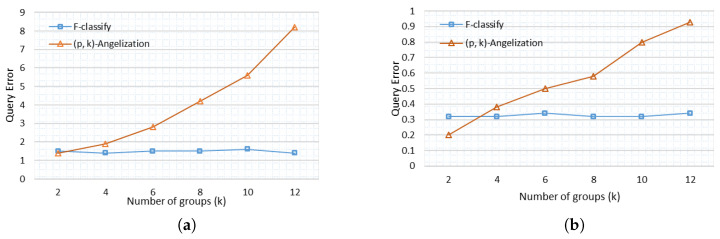
Relative Query Error (RQE) (**a**) RQE for Heart Disease (**b**) RQE for Adults.

For the Heart Disease and Adults data sets, relative query error is plotted against the number of groups in Figure 6a,b. As the group size in F-Classify is not fixed like it is in (p, k) angelization, the relative query error for different group sizes is almost the same in the proposed approach. Furthermore, in the suggested methodology, fuzzy classification is applied for both QIs and SAs, resulting in a relatively low relative query error as compared to (p, k) angelization. Although enhanced generalization is employed for QIs in (p, k) angelization, query error is still greater than in F-Classify.

### 6.6. Execution Time Analysis

**Figure 7:**
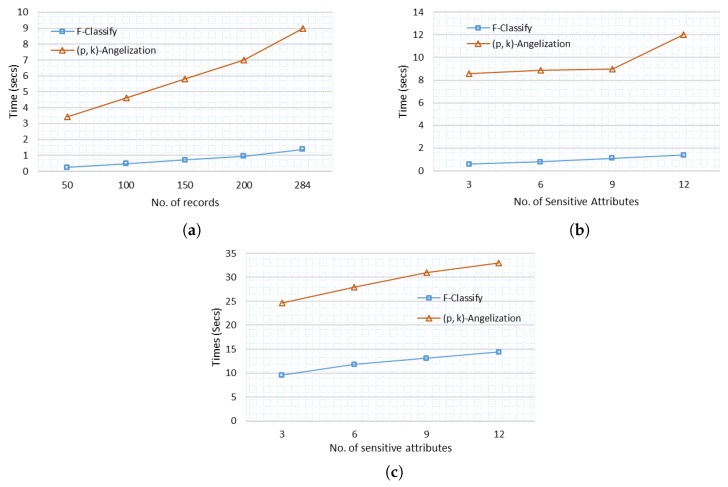
Execution time. (**a**) Execution time with increasing number of records (Heart Disease). (**b**) Execution time with varying number of sensitive attributes (Heart Disease). (**c**) Execution time with varying number of sensitive attributes (Adults).

Variable numbers of records and sensitive attributes are used to compare execution time. The execution time increases as the number of records increases. While the execution time for F-Classify increases slightly as the number of records increases, the execution time for (p, k) angelization increases rapidly as the number of records grows, as illustrated in Figure 7a. When contrast to F-classify, which classifies SAs using an AI algorithm, (p, k) angelization takes longer to execute since it employs a weight calculation technique for each SA. F-Classify has a slight variation in execution time with the increasing number of SAs shown in Figure 7b. The execution time of an adult data set with 40,000 records is presented in Figure 7c, and the pattern is the same: as the number of SAs increases, so does the execution time. In both small and large data sets, (p, k) angelization takes longer to execute than F-Classify.

## 7. Conclusions

This article introduces F-Classify, an AI-based technique for preserving the privacy of multiple sensitive attributes while publishing micro-data. The classification of SAs and QIs in F-Classify is done using rule-based fuzzy classification. Classifying QIs and SAs using fuzzy classification provides for multi-dimensional partitioning with minimal information loss. Attribute disclosure is prevented by classifying SAs into distinct classes so that instead of a fixed number of tuples in each group, each class has a varying number of tuples. Permuting the tables after multi-dimensional partitioning results in a higher level of privacy. Experiments on real-time data sets were conducted, and the results were compared in terms of Query Error, NCP, DCP, and execution. When comparing DCP to (p, k) angelization, it was observed that F-Classify had the lowest DCP value, indicating that records are indistinguishable. Query accuracy is used to assess utility, and it reveals that F-Classify has a lower query error than (p, k) angelization. In comparison to (p, k) angelization, an algorithm’s execution time is quite minimal. We plan to extend this work in the future to include dynamic data publication. Multiple releases with insertion, deletion, and updating of records are a challenge in dynamic data publication.

## Figures and Tables

**Table 1:** Original data table.

Name	Gender	Age	Zipcode	Disease	Treatment	Physician	Symptom	Diagnostic Method
John	M	27	14248	HIV	Antiretroviral therapy (ART)	John	Infection	Blood Test
Ana	F	28	14207	HIV	ART	John	Weight loss	ELISA Test
Richard	M	26	14206	Cancer	Radiation	Alice	Weight loss	MRI Scan
Dave	M	25	14249	Cancer	Chemotherapy	Bob	Abdominal Pain	Chest X-ray
Kate	F	41	13053	Hepatitis	Drugs	Sarah	Fever	Blood test
William	M	48	13074	Phthisis	Antibiotic	David	Fever	Molecular diagnostic methods
Robert	M	45	13064	Asthma	Medication	Suzan	Shortness of breath	Methacholine challenge tests
Olivia	F	42	13062	Obesity	Nutrition control	Steven	Eating disorders	Body mass index (BMI)
Emily	F	33	14248	Flu	Medication	Suzan	Fever	RITD tests
Alec	M	37	14204	Flu	Medication	Eve	Fever	RITD tests
Oliver	M	36	14205	Flu	Medication	Anas	Fever	RITD tests
James	M	35	14248	Indigestion	Medication	Jem	Heartburn	Chest X-ray
Jessica	F	28	14249	Cancer	Chemotherapy	Bob	Abdominal pain	Chest X-ray

**Table 2:** Anonymized table (T*).

P_ID	Age	Zipcode	Group Id	Disease	Treatment	Physician	Symptom	Diagnostic Method
P1	25–28	14206-14249	1	HIV	Antiretroviral therapy (ART)	John	Infection	Blood Test
P2	28–41	13053-14248	2	HIV	ART	John	Weight loss	ELISA Test
P3	25–28	14206-14249	1	Cancer	Radiation	Alice	Weight loss	MRI Scan
P4	25–28	14206-14249	1	Cancer	Chemotherapy	Bob	Abdominal Pain	Chest X-ray
P5	28–41	13053-14248	2	Hepatitis	Drugs	Sarah	Fever	Blood test
P6	33–48	13062-14248	3	Phthisis	Antibiotic	David	Fever	Molecular diagnostic methods
P7	33–48	13062-14248	3	Asthma	Medication	Suzan	Shortness of breath	Methacholine challenge tests
P8	33–48	13062-14248	3	Obesity	Nutrition control	Steven	Eating disorders	Body mass index (BMI)
P9	33–48	13062-14248	3	Flu	Medication	Suzan	Fever	RITD tests
P10	28–41	13053-14248	2	Flu	Medication	Eve	Fever	RITD tests
P12	28–41	13053-14248	2	Indigestion	Medication	Jem	Heartburn	Chest X-ray
P13	25–28	14206-14249	1	Cancer	Chemotherapy	Bob	Abdominal pain	Chest X-ray

**Table 3:** Fuzzy classification of QIs and MSAs.

(a) Classification of QIs (Age-Zipcode)				
**P-ID**	**Age**	**Zip**	**Class**	
P10	[25–33]	[13053-14205]	q-C1	
P5				
P6				
P7	[35–48]	[13053-14205]	q-C2	
P8				
P11				
P1				
P2				
P3	[25–33]	[14206-14249]	q-C3	
P4				
P9				
P13				
P12	[35–48]	[14206-14249]	q-C4	
**(b) Classification of Sensitive Attributes (Symptom-Diagnostic Method)**				
**P-ID**	**Symptom**	**Diagnostic** **Method**	**Class**	
P1 P2 P5	Infection Weight loss Fever	Blood Test Elisa test Blood test	αC1	
P3 P4 P6 P9 P10 P11 P13	Weight loss Abdominal pain Fever Fever Fever Fever Abdominal Pain	MRI Scan Chest X-ray Molecular diagnostic Methods RITD tests RITD tests Chest X-ray	αC2	
P7 P8 P12	Shortness of breath Eating disorders Heartburn	Methacholine challenge tests Body mass index (BMI) Chest X-ray	αC3	
**(c) Classification of Sensitive Attributes (Disease-Treatment-Physician)**				
**P-ID**	**Disease**	**Treatment**	**Physician**	**Class**
P1 P2 P3	HIV HIV Cancer	ART ART Radiation	John John Alice	C1
P4 P5 P13	Cancer Hepatitis Cancer	Chemotherapy Drugs Chemotherapy	Bob Sarah Bob	C2
P6 P7 P9	Phthisis Asthma Flu	Antibiotic Medication Medication	David Suzan Suzan	C3
P8 P10 P11 P12	Obesity Flu Flu Indigestion	Nutritional Control Medication Medication Medication	Steven Eve Anas Jem	C4

**Table 4:** Anonymized QT and MST.

(a) Anonymized Table (QT)					
**P-ID**	**Age**	**Zip**	**Age-Zip Class**	**Physician-Disease-Treatment**	**Symptom-Diagnostic Method**
P10	[25–33]	[13053-14205]	q-C1	C4	αC2
P5 P6 P7 P8 P11	[35–48]	[13053-14205]	q-C2	C2 C3 C4	αC1 αC2 αC3
P1 P2 P3 P4 P9 P13	[25–33]	[14206-14249]	q-C3	C1 C2 C3	αC1 αC2
P12	[35–48]	[14206-14249]	q-C4	C4	αC3
**(b) Anonymized Table (Multiple Sensitive Attribute (MST (1)))**					
**Disease**	**Treatment**	**Physician**	**Class**		
HIV HIV Cancer	ART ART Radiation	John John Alice	C1		
Cancer Hepatitis Cancer	Chemotherapy, Drugs Chemotherapy	Bob Sarah Bob	C2		
Phthisis Asthma Flu	Antibiotic Medication Medication	David Suzan Suzan	C3		
Obesity Flu Flu Indigestion	Nutritional Control Medication Medication Medication	Steven Eve Anas Jem	C4		
**(c) Anonymized Table (Multiple Sensitive Attribute (MST (2)))**					
**Symptom**	**Diagnostic** **Method**	**Class**			
Infection Weight loss Fever	Blood Test Elisa test Blood test	αC1			
Weight loss Abdominal pain Fever Fever Fever Fever Abdominal Pain	MRI Scan Chest X-ray Molecular diagnostic Methods RITD tests RITD tests Chest X-ray	αC2			
Shortness of breath Eating disorders Heartburn	Methacholine challenge tests Body mass index (BMI) Chest X-ray	αC3			

**Table 5:** Comparison of MSAs based approaches.

	Privacy Models	Evaluation	Attacks	Utility
[22]	Slicing	It was intended for high dimensional data, but it has failed and has given original tuples when multiple tuples have identical SAs and QIDs.	Skewness, sensitivity, and similarity attacks	Loss of information
[7]	Slicing and anatomization	The proposed approach has a very complex solution. It publishes multiple tables, and also has greater execution time.	Demographic knowledge attack	Loss of information
[38]	Multiple column multiple attributes slicing	The proposed approach is for the MSAs anonymization, and QIs are overlooked. In case of 1:M occurrence of record, it shows incorrect results.	Skewness attacks, similarity attacks, and sensitivity attacks	Loss of information
[9]	SLOMS	Proposed approach released several tables with information loss. The correlation among MSA was also removed in this approach.	Demographic knowledge attack	Loss of information
[24]	Multi-sensitive bucketization with clustering	The approach only worked with numerical data if the consequence suppression rate is low.	-	Information loss is less
[25]	MSA(α,l)	The approach used generalization with suppression and anatomy. It caused the utility to decrease.	-	High information loss
[27,39]	(α,l), Anatomy, generalization, and suppression	Decrease in utility due to suppression of SA values.	-	Loss of information
[28]	Rating	SAs are generalized.	Association privacy attack	Loss of information
[29]	Decomposition	The proposed approach preserves privacy by assuring diversity in MSAs, as a consequence it activated information loss.	Similarity and skewness privacy attacks	Loss of information is high
[30]	Decomposition plus	Noise is added in proposed method, resulting in loss of utility. Attribute and identity disclosure are also not prevented in this approach.	Similarity and skewness privacy attacks	Loss of information is high
[31]	ANGELMS	There is a zero correlation between MSAs and QIDs in this approach, results in high information loss.	Sensitivity, similarity, and skewness privacy attacks	Loss of information is high.
[32]	P+ sensitive t-closeness	It assigns sensitivity level to each SA in such a way that each group contains at least p-distinct sensitivity levels. It also generalizes the QIs.	-	Loss of information
[40]	P-cover k-anonymity	It generalizes QI values to ensure privacy, it also ensures the MSA P-diversity constraint between MSA. It avoids membership, identity and attribute disclosures.	Sensitivity, skewness, and similarity privacy attacks	Loss of information.
[8]	(p, k)-angelization	The proposed approach preserves the privacy of MSAs using weight calculations. Weight calculation takes additional execution time and hence resulted into higher execution time.	-	Loss of information.

**Table 6:** Summary of notations.

Symbol	Description
DT	Data Table
ST	Subset of quasi attributes and sensitive attributes in ST
QA	Quasi identifier
SA	Sensitive attribute
MSAs	Multiple sensitive attributes
Class	Classes of quasi attributes and sensitive attributes
q-C	Quasi identifier class
sa-C	Sensitive attribute class
m	Number of data attributes
n	Number of tuples
(lv)	Linguistic variables
μ	Membership function for linguistic variables
Rules	Fuzzy
η	Number of member ship functions for l$v_{m}$
α	Number of fuzzy rules
γ	Number of attributes in one subset
Qc T	Quasi attributes class based tables
Sc T	Sensitive attributes class based tables
Anonymize T	Anonymize table of quasi and sensitive attributes
QT	Quasi identifier table
MST	Multiple sensitive attribute tables

**Table 7:** Types used in HLPN for F-Classify algorithm.

Types	Description
Tp${}_{m}$	*m* tuples in Data Table
Dq	Subset of quasi-identifier
Ds${}_{i}$	Multiple Subsets of sensitive attribute values
Qc	Class for quasi-identifiers
Sci	Multiple classes for sensitive attributes
LV${}_{m}$	Linguistic variables for *m* attributes
R${}_{\mu }$	mu number of fuzzy rules
PID	Patient identifier in Data Table
Q	Group of quasi identifiers
C${}_{q}$	Quasi identifier classes
C${}_{s}$	Sensitive attribute classes
SA${}_{i}$	Multiple number of sensitive attribute Tables

**Table 8:** Mapping of data types on places.

Types	Description
φ(DT)	P (PID × Tp${}_{m}$)
φ(ST)	P (Dq × Ds${}_{i}$)
φ(Class)	P (Qc × Sc${}_{i}$)
φ(L−Variable)	P (L V${}_{m}$)
φ(MF)	P (mf${}_{i}$)
φ(Rules)	P (R${}_{\mu }$)
φ(QcT)	P (PID × Q x C${}_{q}$)
φ(ScT)	P (PID × SA${}_{i}$ × C${}_{s}$ )
φ(AnonymizeT)	P ((PID × Q × C${}_{q}$ x C${}_{s}$)
φ(AnonymizeQT)	P ((Q × C${}_{q}$)
φ(MST)	P ((SA${}_{i}$ × C${}_{i}$)
